# Supplementary material for: Hospitalizations for Acute Salicylate Intoxication in the United States
Source: J Clin Med. 2020 Aug 14;9(8):2638. doi: 10.3390/jcm9082638 (PMC7465677; doi:10.3390/jcm9082638)
Supplement: Supplementary file 1 [file jcm-09-02638-s001.pdf]

Table S1 ICD 9 CM codes

Salicylate poisoning	965.1
Smoking	305.1, 649.0, 989.84
Alcohol drinking	291.0, 291.1, 291.2, 291.3, 291.4, 291.5, 291.8, 291.81, 291.82, 291.89, 303.00-303.03, 303.90-303.93, 305.00-305.03
Anemia	283.00-285.9
Obesity	278.0, 278.00, 278.01, 649.10– 649.14, 793.91, V85.30–V85.4, V85.54
Diabetes Mellitus	249.00–249.31, 250.00–250.33, 648.00–648.04, 249.40–249.91, 250.40–250.93, 775.1
Hypertension	401.1, 401.9, 642.00–642.24, 401.0, 402.00– 405.99, 437.2, 642.10–642.24, 642.70–642.94
Dyslipidemia	272.xx
Congestive heart failure	428.xx
Chronic kidney disease	585.1, 585.2, 585.3, 585.3, 585.4, 585.5, 585.6, 585.9
Liver cirrhosis	456.xx, 567.23, 571.xx, 789.59
Atrial flutter/fibrillation	427.31, 427.32
Acute coronary syndrome	410.xx, 411.xx
Chronic ischemic heart disease	412.xx, 413.xx, 414.xx
Ventricular arrhythmia /Cardiac arrest	427.1, 427.41, 427.5
Invasive mechanical ventilation	96.70-96.73
Non-invasive ventilation	93.90
Hemodialysis	39.95, v45.1, v56.0, v56.1
Peritoneal dialysis	54.98, v56.2, v56.32
Gastric lavage	96.07-96.08, 96.33-96.36
Blood transfusion	99.00-99.07
Hyponatremia	276.1
Hypernatremia	276.0
Hypokalemia	276.8
Hyperkalemia	276.7
Metabolic acidosis	276.2
Metabolic alkalosis	276.3
Volume depletion disorder	276.5, 276.51, 276.52
Hypoglycemia	250.3, 250.8, 251.0, 251.1, 251.2, 270.3, 775.0, 775.6, 962.3
Gastrointestinal bleeding	456.0, 456.20, 530.82, 531.00-531.41, 531.60, 531.61, 532.00-532.41, 532.60-532.61, 533.00-533.41, 533.60, 533.61, 534.00-534.41,534.60, 569.3, 578.0, 578.1, 578.9
Seizure	345.00-345.91, 780.3-780.39, 89.14

Rhabdomyolysis	728.88
Sepsis	003.1, 003.21, 020.2, 022.3, 036.0, 036.1, 036.2, 036.3, 036.42, 038.0, 038.10, 038.11, 038.19, 038.2, 038.3, 038.4, 038.40, 038.41, 038.42, 038.43, 038.44, 038.49, 038.8, 038.9, 098.82, 098.84, 098.89, 112.5, 112.81, 112.83, 115.04, 115.14, 115.94, 117.9, 785.52, 790.7, 995.92
Acute kidney injury	584, 584.5, 584.6, 584.7, 584.8, 584.9 (exclude 585.5, 585.6)
Respiratory failure	518.81, 518.82, 518.85, 786.09, 799.1, 96.7, 96.70, 96.71, 96.72
Circulatory failure	458.8, 458.9, 785.5, 785.50, 785.51, 785.52, 785.59, 796.3
Liver failure	570, 572.2, 573.3, 573.4
Neurological failure	293, 293.0, 293.1, 293.8, 293.81, 293.82, 293.83, 293.84, 293.89, 293.9, 348.1, 348.3, 348.30, 348.31, 780.01, 780.09, 48.39, 89.14
Hematological failure	286.6, 286.7, 286.9, 287.49, 287.5
